# Integration of HIV care into maternal health services: a crucial change required in improving quality of obstetric care in countries with high HIV prevalence

**DOI:** 10.1186/1472-698X-13-27

**Published:** 2013-06-10

**Authors:** Farai D Madzimbamuto, Sunanda Ray, Keitshokile D Mogobe

**Affiliations:** 1 Department of Anaesthesia, University of Botswana School of Medicine, Gaborone, Botswana; 2 Department of Public Health, University of Botswana School of Medicine, Gaborone, Botswana; 3 University of Botswana School of Nursing, Gaborone, Botswana; 4 University of Zimbabwe College of Health Sciences, Mazowe Street, Belgravia, Harare, PO Box A178, Avondale, Harare, Zimbabwe

**Keywords:** Maternal mortality, HIV, Health systems, Quality of obstetric care, Southern Africa, Integrated HIV and maternity services

## Abstract

**Background:**

The failure to reduce preventable maternal deaths represents a violation of women’s right to life, health, non-discrimination and equality. Maternal deaths result from weaknesses in health systems: inadequate financing of services, poor information systems, inefficient logistics management and most important, the lack of investment in the most valuable resource, the human resource of health workers. Inadequate senior leadership, poor communication and low staff morale are cited repeatedly in explaining low quality of healthcare. Vertical programmes undermine other service areas by creating competition for scarce skilled staff, separate reporting systems and duplication of training and tasks.

**Discussion:**

Confidential enquiries and other quality-improvement activities have identified underlying causes of maternal deaths, but depend on the health system to respond with remedies. Instead of separate vertical programmes for management of HIV, tuberculosis, and reproductive health, integration of care and joint management of pregnancy and HIV would be more effective. Addressing health system failures that lead to each woman’s death would have a wider impact on improving the quality of care provided in the health service as a whole. More could be achieved if existing resources were used more effectively. The challenge for African countries is how to get into practice interventions known from research to be effective in improving quality of care. Advocacy and commitment to saving women’s lives are crucial elements for campaigns to influence governments and policy -makers to act on the findings of these enquiries. Health professional training curricula should be updated to include perspectives on patients’ rights, communication skills, and integrated approaches, while using adult learning methods and problem-solving techniques.

**Summary:**

In countries with high rates of Human Immunodeficiency Virus (HIV), indirect causes of maternal deaths from HIV-associated infections now exceed direct causes of hemorrhage, hypertension and sepsis. Advocacy for all pregnant HIV-positive women to be on anti-retroviral therapy must extend to improvements in the quality of service offered, better organised obstetric services and integration of clinical HIV care into maternity services. Improved communication and specialist support to peripheral facilities can be facilitated through advances in technology such as mobile phones.

## Background

Human Immunodeficiency Virus [HIV] infection is now the most important condition contributing to maternal deaths in the southern Africa region, with HIV-positive women in South Africa more likely to die of any underlying cause in pregnancy than HIV-negative women [1-4]. The obstetric care of HIV-positive women and the literature surrounding it have largely focused on preventing HIV transmission to infants. Pregnancy outcomes are often stated in terms of the survival of the infant rather than survival of the mother. However, advocates in Africa are calling for all pregnant women with HIV to be on anti-retroviral therapy [ART], anticipating that this intervention will reduce maternal deaths in the next few years, [5-8] despite emerging evidence that infant outcomes may not be so good [9]. This paper argues that provision of ART to all pregnant women in high HIV-prevalent areas is not enough. Much more attention must be focused on integration of clinical HIV care into maternity services, with problem-solving audits on deaths, leading to improved quality of care. Finding the resources to do this is a pre-requisite to reducing maternal deaths.

## Discussion

### Integration of HIV and maternity services

One consequence of the impact of HIV on maternal mortality is that indirect causes of death now exceed direct obstetric causes, with non-pregnancy related infections predominating [1,5]. Tuberculosis is often under-diagnosed and treated late. Women are presenting to maternity services already ill with HIV-related problems, often with complex conditions that are outside the expertise of antenatal care. Obstetric services have to reorient to take account of these changes in countries with high HIV-prevalence. The skills birth attendants need for these situations include screening for opportunistic infections. A chronic cough in pregnancy could mean tuberculosis or heart failure; a severe headache may herald meningitis as well as hypertension. In countries where HIV is managed in vertical programmes through separate infectious disease clinics, pregnant women may attend parallel services for different aspects of their condition. Women in late pregnancy may be admitted to gynaecology wards when they are suffering from medical conditions such as pneumonia or meningitis, with little input from physicians. Joint management between the two specialties is essential with clear delivery plans rather than women delivering unattended on medical wards, or having their tuberculosis undiagnosed and untreated on a gynaecology ward. More intensive medical inputs would also be needed for cardiac, respiratory and metabolic disorders in pregnancy which are often HIV-related. In district hospitals with less specialist input, medical officers, nurses or mid-level health workers [clinical officers, clinical associates, or medical assistants] will need to cover both. They will need training on how HIV impacts pregnancy, to treat within their limitations and to transfer complex cases early. Improved communication and specialist support to peripheral hospitals is crucial and can be facilitated through advances in technology, such as mobile phones and email. Where a pregnant woman’s death from AIDS is inevitable, palliative care to enable a dignified pain-free death must be instituted, with a management plan for delivering her infant where possible. Termination of pregnancy on medical grounds for the mother, legally permissible in most southern African countries, may also be considered where it may make a difference to an HIV-positive woman’s clinical condition.

Conventional thinking that most obstetric complications cannot be predicted or prevented through early detection and risk-assessment in antenatal care now mainly applies to direct causes of maternal deaths [10]. With HIV and indirect causes the death is often a complication of underlying disease which can be detected in the antenatal period if screening and follow-up for opportunistic infections are carried out. Patients with HIV are at greater risk of sepsis complicating the delivery, whether vaginal or by Caesarean Section [11]. Data from southern Africa show that the greater proportion of maternal deaths in HIV-positive women occur in the postpartum period, mainly from AIDS [11]. Many of these women are still inpatients and need inter-specialist care in postnatal wards to prevent deterioration and death. Recommendations that the first postnatal review be reduced from six to two weeks have now been revised to less than a week for all pregnant women [12]. Putting all pregnant women with HIV on ART, regardless of their immune status, may mean that they do not present in pregnancy in such a sick condition. These women still need careful monitoring of their clinical condition, in a continuum of care through antenatal care, delivery and postnatal care, with special alertness to possible complications of antiretroviral drugs [1,13].

### Quality improvement interventions

International variations in the impact of pregnancy on survival of HIV-positive women or their progression to AIDS, have been attributed to quality of care, access to treatment and healthcare [3]. The main direct obstetric causes of death: haemorrhage, hypertension and sepsis, are usually the immediate events leading to death. In- depth investigation, using a critical incident or root-cause analysis, reveal a number of factors that contribute to why each woman died [14]. Trying to tackle these with single interventions: a guideline on eclampsia perhaps or a blood donation campaign, is tinkering at the edges of the problem. Maternal deaths are red flags for weaknesses in health systems: failure to recognize the seriousness of complications, lack of urgency in dealing with these cases, and the need for more aggressive and prompt action. Inadequate senior leadership, poor communication and low staff morale are cited repeatedly in explaining low quality of care [15]. Emergency obstetric care training, a practical experiential approach, has been successfully implemented in many African countries, but the training needs for prevention of maternal deaths are wider than this programme.

Improved organisation of obstetric services, rather than changing social determinants, were instrumental in reducing maternal deaths in Europe and the USA after the mid-1930s. Considerable progress was made over a span of 15–20 years, a similar time period to that planned for achievement of the Millennium Development Goals. Having well-trained and well-supervised midwives was a key feature of this progress, but also determination on the part of health professionals to address underlying causes [16]. Confidential enquiries in the UK, with reports published triennially since 1952 which detailed events leading to each maternal death, contributed to the knowledge of where changes had to be made and were emulated as models in other countries [16,17]. Key outputs were more focused training, supervision, guideline development and implementation, and outcome monitoring. Quality improvement activities, clinical audits, checklists and care bundles that have appreciably improved outcomes in critical care and surgery, have also resulted in improved outcomes in obstetric services and should be scaled up [18,19]. Confidential enquiries are potentially powerful instruments of change, but only if the recommendations made are acted on. Staff shortages are a considerable constraint but even countries that are better resourced such as South Africa and Botswana are struggling to meet their targets. More could be achieved if existing resources were used effectively. There is now an impressive body of knowledge addressing the health human resource deficit in Africa, how to raise morale, which incentives work, task-shifting, supportive supervision, mentoring teams and so on [20]. The challenge for many African countries is how to get into practice the whole raft of interventions known from research to be effective in improving quality of care.

A health systems approach takes account of the whole interconnected and multifactorial network of workforce, logistics, infrastructure, finance and data management that work together to deliver health services, rather than disease-focused or single-issue perspectives. Proponents of such approaches warn against concentrating resources in vertical programmes, leaving other areas with fewer skilled staff, and not managing the interface between conditions. Duplication of tasks, competition for skilled staff, uncoordinated training and conflicts in reporting to different authorities may result from vertical programmes operating within one health system [21]. Integration of HIV counseling and testing has been advocated for other sexual and reproductive health services such as family planning and prevention of mother to child transmission [22]. Joint management of clinical HIV treatment and care with maternity services in high HIV-prevalent countries is now essential. Integration of HIV into antenatal services and use of HIV resources to strengthen health infrastructure in Mozambique, for example, led to “system efficiency” through reduced workforce gaps, improved supervision, patient flows and coordination between laboratories, pharmacies and clinics, in ways that benefited all programmes [23].

### Advocacy and training

Leadership and advocacy, for instance from medical and nursing associations, are critical to make sure politicians prioritise resources for these recommendations, but also for changes in attitude of the health professionals involved. Alliances with community organizations and activists are needed to raise these concerns to political platforms [24]. Advocacy and commitment to the humanitarian principle of preventing women’s deaths in childbirth were key ingredients found in a study of political priority given to this issue in several countries [25]. The author suggests that national health advocates are more likely to be effective if they coalesce into unified policy communities and networks that use their authority to influence policy-makers and governments to act. Leaders must also be presented with clear policy alternatives that are proven to be effective and are feasible to carry out [25]. These alternatives will arise from painstaking reviews on the underlying reasons for each woman’s death. The foundation for improving maternal services is to establish a “No blame, no shame” culture with a constructive, morale-building, inclusive approach and motivational leadership at all levels of policy and care. Curricula for training all health professionals must be re-orientated to include patients’ rights perspectives, and use of adult learning approaches, including communication skills, evidence reviews and team-building that accompanies problem-based learning methods. Development of a clinical audit culture will help health professionals learn from their mistakes rather than being scolded for them, provide feedback on good practice at all opportunities, promote regular reviews to monitor and update guidelines in line with current scientific knowledge and evidence, and encourage improved documentation and safe-keeping of case records for review purposes. Quality improvement of maternal health services will not only prevent avoidable deaths but also benefit the health system as a whole.

Failure to reduce preventable maternal deaths represents a violation of women’s right to life, health, non-discrimination and equality. As signatories to international human rights conventions and to the Millennium Development Goals, nation states are obliged to provide the necessary services of sufficient quality to prevent these deaths. The Convention on the Elimination of All Forms of Discrimination against Women [CEDAW] requires that governments ensure that women have access to “appropriate services in connection with pregnancy, confinement and the postnatal period, granting free services where necessary, as well as adequate nutrition during pregnancy and lactation” [article 12.2] [26].

## Summary

The care of HIV-positive pregnant women has become one of the key challenges to meeting the MDG 5 in Southern Africa. A prerequisite to reducing maternal deaths in countries with high prevalence of HIV infection is to improve quality of care and integrate HIV care into maternal health services. Historical precedence in better-resourced countries has shown that efficient organization of obstetric services, with training of birth attendants and follow-up of deaths through confidential enquiries, has been successful in reducing deaths over a period of 15–20 years. Valuing women’s lives, recognizing their right to safe maternity services, and investment in their health cannot be a quick fix, but has to be a long-term investment, commitment and sustainable process.

## Abbreviations

ART: Anti-retroviral therapy; HIV: Human immunodeficiency virus; MDG 5: Millennium development goal 5; CEDAW: Convention on the elimination of all forms of discrimination against women.

## Competing interests

All authors declare that they have no competing interest.

## Authors’ contributions

FD, SR & KDM conceived of the paper and initial references. FD and SR wrote the first draft and subsequent amendments. All authors contributed, read and approved final manuscript.

## Pre-publication history

The pre-publication history for this paper can be accessed here:

http://www.biomedcentral.com/1472-698X/13/27/prepub
